# Student mental health during the first two years of the COVID- 19 pandemic

**DOI:** 10.1192/j.eurpsy.2022.1367

**Published:** 2022-09-01

**Authors:** E. Charbonnier, S. Le Vigouroux, C. Puechlong, L. Montalescot, A. Goncalves

**Affiliations:** APSY, Université De Nîmes, Nimes, France

**Keywords:** coping, mental health, UNIVERSITY, Covid-19

## Abstract

**Introduction:**

The COVID-19 pandemic have had deleterious effects on mental health of students. Authors suggest that the psychological effects will persist long after COVID-19 has peaked, but we have no data to confirm this.

**Objectives:**

Objective: The objective of this study is to compare clinical issues (concerns, anxiety and depression symptoms) and adjustment (coping strategies) in French university students during different phases of the COVID-19 pandemic in 2020 and 2021 (during two periods of lockdown and two periods after lockdown)

**Methods:**

Method: Data were collected anonymously at four timepoints: during France’s first national lockdown (23 April- 8 May 2020; n_T1_ = 1294); during the period after lockdown (9‑23 June 2020; n_T2_ = 321); 1 year after the first lockdown, which was also a lockdown period (23 April- 8 May 2021; n_T3_ = 2357); and 1 year after the first unlockdown, which was also a unlockdown period (9‑23 June 2021, n_T4_ = 1174). The following variables were measured: concerns, coping strategies, anxiety and depressive symptoms.

**Results:**

In 2021, students have significantly higher levels of anxiety and depressive symptoms than in 2020, and this is even more pronounced during the lockdown periods. For example, 44.1% had probable anxiety symptoms in the 2021 lockdown, compared to 33% in the 2020 lockdown. In the unlockdown periods, the rates are 21.7% in 2020 and 26.4% in 2021.

**Conclusions:**

Our results suggest that university students, known to be a vulnerable population with significant mental health deterioration, have become even more vulnerable with the COVID-19 pandemic.

**Disclosure:**

No significant relationships.

